# Optimal Use of Vaccines for Control of Influenza A Virus in Swine

**DOI:** 10.3390/vaccines3010022

**Published:** 2015-01-30

**Authors:** Matthew R. Sandbulte, Anna R. Spickler, Pamela K. Zaabel, James A. Roth

**Affiliations:** Center for Food Security and Public Health, College of Veterinary Medicine, Iowa State University, Ames, IA 50011, USA; E-Mails: sandbult@iastate.edu (M.R.S.); spickler@iastate.edu (A.R.S.); zaabelp@iastate.edu (P.K.Z.)

**Keywords:** influenza A virus in swine, vaccines, immune response, surveillance, veterinary diagnostics

## Abstract

Influenza A virus in swine (IAV-S) is one of the most important infectious disease agents of swine in North America. In addition to the economic burden of IAV-S to the swine industry, the zoonotic potential of IAV-S sometimes leads to serious public health concerns. Adjuvanted, inactivated vaccines have been licensed in the United States for over 20 years, and there is also widespread usage of autogenous/custom IAV-S vaccines. Vaccination induces neutralizing antibodies and protection against infection with very similar strains. However, IAV-S strains are so diverse and prone to mutation that these vaccines often have disappointing efficacy in the field. This scientific review was developed to help veterinarians and others to identify the best available IAV-S vaccine for a particular infected herd. We describe key principles of IAV-S structure and replication, protective immunity, currently available vaccines, and vaccine technologies that show promise for the future. We discuss strategies to optimize the use of available IAV-S vaccines, based on information gathered from modern diagnostics and surveillance programs. Improvements in IAV-S immunization strategies, in both the short term and long term, will benefit swine health and productivity and potentially reduce risks to public health.

## 1. Introduction

Influenza A virus in swine (IAV-S) is considered one of the most important infectious disease agents affecting North American swine [1]. The financial impact of IAV-S to the pork industry comes mainly from reduction in the growth rate of infected pigs [2]. Additionally, IAV-S is a zoonotic pathogen which can be transmitted between people and pigs with consequences to public health. Farm workers or other persons in contact with livestock may become infected with IAV-S or, conversely, they may transmit human IAV to swine [3,4]. It is possible for an IAV-S strain to be adapted to the human host well enough to spread between humans and initiate a pandemic. A version of this occurred in 2009, leading to the H1N1 influenza pandemic. During that time, public misperceptions about the safety of eating pork caused economic losses to the US industry estimated at over $1 billion [5].

Considering the important consequences of IAV-S, effective control measures are highly desirable. A range of farm management practices can help limit the circulation of IAV-S in herds, including the use of vaccines in sows, nursery pigs, or finishers. Swine veterinarians and producers are likely aware that IAV-S vaccines sometimes have disappointing efficacy in the field [6]. However, under the right conditions vaccines can reduce or eliminate transmission of IAV-S in herds [7,8]. Successful IAV-S vaccination can improve herd health and lower the risk of transmission to other species, including humans. This document was developed to provide veterinarians with a scientifically-based overview of IAV-S biology, immunity to the virus, vaccines currently available, and technologies that show promise for the future. It also describes strategies to optimize the use of available IAV-S vaccines for a given herd, with the aid of modern diagnostics and surveillance programs.

## 2. Influenza A Virus Structure and Function

Influenza A viruses (family Orthomyxoviridae) are highly variable, enveloped viruses with negative-sense, single-stranded, segmented RNA genomes. Most influenza A viruses circulate among birds, particularly species that live in aquatic environments [9,10]. Some strains are adapted for efficient replication and sustained transmission in particular mammalian species, including humans and swine [9,11,12,13,14]. Once a virus has become adapted to the swine host, it is considered to be IAV-S, regardless of its ancestral origins.

### 2.1. Roles and Functions of Influenza A Virus Proteins

**Figure 1:**
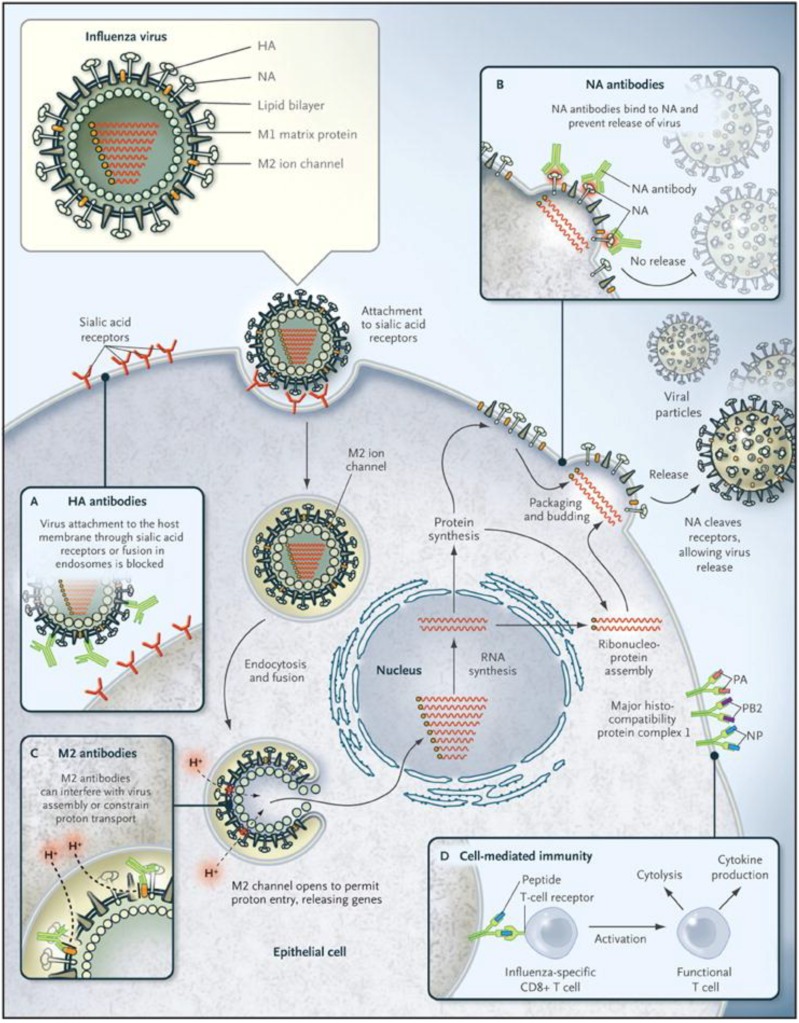
Influenza virus infection cycle. Basic structural features of an influenza virus are diagrammed in the top left corner. Infection begins with the binding of hemagglutinin (HA) proteins to receptor molecules on the cell surface. The cycle is completed when new particles, each containing eight RNA segments, bud off from the cell membrane. Neuraminidase (NA) protein cleaves the bonds between HA and sialic acid molecules, allowing new virus to disperse. Boxes labeled **A**–**D** indicate points in the cycle that may be inhibited by antibodies or T cells. (Figure used with permission from the New England Journal of Medicine, Linda C. Lambert and Anthony S. Fauci, Influenza Vaccines for the Future, Vol. 363:2039. © 2010 Massachusetts Medical Society).

The influenza A virus genome consists of eight segments, which encode for at least 12 proteins (Table 1) [15,16]. Three of these proteins—the viral hemagglutinin (HA), neuraminidase (NA), and matrix 2 (M2) proteins—are incorporated into the envelope of the virus (Figure 1). The HA and NA are glycoproteins with stem and head structures that protrude from the surface of the virus. The HA is the most abundant of the envelope proteins, up to 80% of the total [15].

**Table 1:** Influenza virus proteins.

Abbreviation	Protein	Location	Significance in IAV-S Vaccines
HA	Hemagglutinin	Envelope	Subtype of HA in inactivated virus vaccine must match challenge virus. Vaccines with more closely related HA are more likely to be protective.
NA	Neuraminidase	Envelope	NA content in vaccines is not standardized, but studies indicate a role in protection [17,18,19,20].
M2	Matrix 2	Envelope	Experimental vaccines were directed against highly conserved M2 [21,22].
M1	Matrix 1	Internal	
NP	Nucleoprotein	Internal	
NS1	Nonstructural protein 1	Nonstructural	Gene deletion in NS1confers attenuation in a candidate vaccine [23,24].
NS2/ NEP	Nonstructural protein 2/Nuclear export protein	Nonstructural or internal	
PA	Polymerase acidic protein	Internal	
PB1	Polymerase basic protein 1	Internal	Mutations in PB1 and PB2 confer attenuation in a candidate vaccine [25].
PB1-F2		Internal	
PB2	Polymerase basic protein 2	Internal	Mutations in PB1 and PB2 confer attenuation in a candidate vaccine [25].

#### 2.1.1. Influenza A Virus Hemagglutinin

The HA protein is responsible for binding influenza virions to host cells [14,15]. While binding is complex and is still incompletely understood [26,27], the HA interacts initially with sialic acids that are linked to host cell proteins (Figure 1) [15,26,27]. Influenza A viruses adapted to either people or swine tend to preferentially bind to sialic acid receptors that have α(2,6) linkages with the carbohydrate moiety [26,27,28]. In contrast, most avian influenza viruses have a binding preference for α(2,3) linked sialic acids. The binding preference of each influenza virus tends to match the predominant receptors in the host tissues where it normally replicates. Most of the sialic acid receptors in the nasal epithelium, trachea and bronchi of the human respiratory tract are of the α(2,6) linked form [26]. The absence of α(2,3) sialic acid linkages in the upper respiratory tract is thought to limit the binding and person-to-person transmission of avian influenza viruses. The types of influenza virus receptors in the pig are still incompletely understood. One widely quoted study reported that both α(2,3) and α(2,6) linked sialic acids occur in the upper respiratory tract of swine [29]. Some newer studies suggest that the pattern may instead resemble that of humans [28,30,31]. Once the influenza virion has bound to the cell surface, it enters the host cell in an endosome [15]. The acidic pH in the endosome triggers a conformational change in the HA, which results in the fusion of the viral and endosomal membranes.

#### 2.1.2. Other Influenza Surface Proteins: Neuraminidase and Matrix 2

The function of the influenza A virus NA protein is to cleave sialic acids on host cells, allowing newly made virions to be released efficiently from the infected cell (Figure 1) [14,15]. This protein is required for influenza A viruses to spread efficiently [14], and antibodies to it are likely to impede the spread of virus to uninfected cells. The enzymatic activity of the NA might also facilitate the infection of cells, by helping the virion penetrate the blanket of mucus in the respiratory tract or the glycocalyx surrounding the cell [32].

The transmembrane matrix 2 (M2) protein is a proton-selective ion channel, and is required for efficient uncoating of influenza A viruses [14]. M2 acidifies the viral core after the virus enters the host cell, allowing the viral ribonucleoprotein complexes (vRNPs) containing the gene segments to be released into the cytoplasm (Figure 1) [15].

#### 2.1.3. Internal Proteins of Influenza A Viruses

The internal proteins of influenza A viruses have structural roles, are involved in nucleic acid replication, alter antiviral responses in the host cell, and influence virulence [14,15,16,33]. Matrix 1 (M1) is an abundant protein that forms the internal matrix of the virion [15,33]. During replication, it is thought to be important in virus assembly and budding [14]. The viral ribonucleoprotein complexes, contained within the matrix, consist of viral RNA wrapped around the nucleoprotein (NP) [15,33]. The vRNPs associate with the three polymerase proteins, polymerase acidic protein (PA), polymerase basic protein 1 (PB1) and polymerase basic protein 2 (PB2) [33]. Once the virus uncoats, the vRNPs are transported to the nucleus of the host cell, where the viral RNA-dependent RNA polymerase carries out replication of the genome. Nuclear export protein has an important role in exporting newly made viral RNA from the nucleus [14,33]. The nonstructural protein NS1 is a multifunctional protein, which has been implicated in the inhibition of interferon-mediated host defenses [33,34,35]. An additional protein, PB1-F2, can be found in either a truncated or full length form in different influenza A virus strains [16]. The full length form of PB1-F2 does not seem to be necessary for virus function, and truncation is common in swine H1N1 viruses [16]. This protein appears to modulate immune responses and may increase pathogenicity in some virus/host combinations [16,36].

### 2.2. Influenza A Virus Subtypes

The HA and NA proteins are very diverse, and this diversity is used to classify influenza viruses into subtypes. Currently, at least 18 hemagglutinin antigens (H1 to H18) and nine neuraminidase antigens (N1 to N9) have been recognized [10,37]. Most HA types (H1 through H16) occur in avian influenza viruses, but H17 and H18 were discovered and found exclusively in bats to date [37]. In contrast, only a limited number of subtypes have adapted to circulate in any mammalian species. Currently, most of the influenza A viruses in North American swine populations belong to the subtypes H1N1, H1N2 and H3N2, although other variants (e.g., H3N1 and H2N3) have occasionally been detected (Section 2.4) [38,39].

### 2.3. Generation of Diversity in Influenza A Viruses

**Figure 2:**
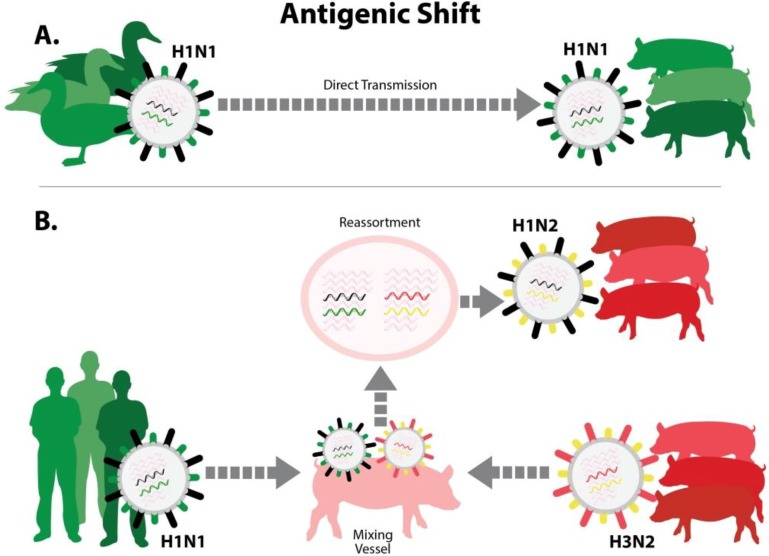
Antigenic shift. There are two ways that an influenza virus with new antigenic properties may enter the pig population. (**A**) Virus that was previously adapted to another animal host, such as avian species, enters pigs and adapts to circulate efficiently in swine. The diagram portrays the inter-species transmission of an avian H1N1 virus, which became established in European swine populations; (**B**) Virus previously adapted to another host, such as birds or humans, co-infects a pig along with a common swine-adapted strain. This can lead to gene reassortment, producing a new “reassortant” virus that contains an HA and/or NA antigenically different from those that previously circulated in swine. The diagram portrays reassortment between human seasonal H1N1 and swine H3N2 viruses. In both (**A**) and (**B**), the swine population lacks antibodies to important surface proteins of the new virus.

Diversity in influenza A viruses can be produced by mechanisms that fall under two categories, “antigenic shift” and “antigenic drift”. The least complicated form of antigenic shift occurs if pigs (or another host population, such as humans) are infected with an influenza A virus that transmits “whole” from another species, such as a bird or a human [13,40] (Figure 2A). This type of event generally requires a period of adaptation, during which the virus gains efficiency at replicating in the new host. Another form of antigenic shift is a consequence of the unique influenza virus genome, which has eight gene segments. This makes possible the exchange of gene segments between two influenza viruses that infect the same individual animal (called “gene reassortment”). Reassortment generates novel viruses that express proteins in new combinations (Figure 2B), including variants that contain either a new HA, a new NA, or both. Antigenic shift variants, whether resulting from whole-virus interspecies transmission or gene reassortment, can completely evade the pre-existing immunity in the host population.

**Figure 3:**
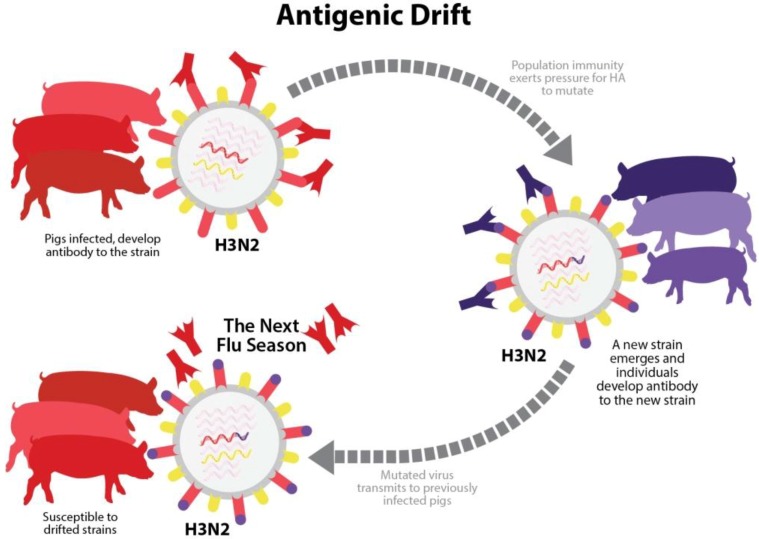
Antigenic drift. Over time, random mutations in HA and NA genes of an influenza A virus in swine (IAV-S) strain may cause significant changes in antigenic properties. A swine herd with population immunity to IAV-S has neutralizing antibodies specific to a strain that was previously encountered through infection or vaccination. However, if antigenic drift produces a new variant strain that pre-existing antibodies in the herd are unable to neutralize, the pigs become susceptible to reinfection.

More gradual changes in the HA and NA, or “antigenic drift”, result from accumulated mutations in the HA or NA proteins of the virus (Figure 3). The influenza A virus polymerase is error-prone, facilitating such mutations. Antigenic drift also allows the virus to evade pre-existing immune responses, although this occurs more slowly than with antigenic shifts.

The internal proteins of influenza A viruses tend to accumulate fewer mutations than the HA and NA. Nevertheless, there is some variability, which is thought to contribute to the adaptation of the virus to a host species [26]. One particular combination of internal viral genes, called the TRIG cassette (Section 2.4.1), has become widespread in North American influenza viruses of swine, and seems to promote their acquisition of new HA and NA proteins [39].

### 2.4. Subtypes of Influenza A Virus in Swine

#### 2.4.1. North America

The populations of IAV-S found in North America are currently very diverse. They include H3N2 viruses, H1N1 and H1N2 viruses (Table 2), and occasionally other subtypes with a limited distribution.

**Table 2:** Major IAV-S lineages that have been found among pigs in North America.

Virus	Origin of HA	Origin of NA	Internal Genes (Typical)
Classical H1N1	swine adapted (1918)	swine adapted (1918)	swine adapted (1918)
Reassortant H1N1 (rH1N1)	classical H1N1	classical H1N1	TRIG
Human-like H1N1	human IAV	human IAV	TRIG
2009 Pandemic H1N1	classical H1N1	Eurasian IAV-S	TRIG lineage; M gene from Eurasian virus
H1N2	classical H1N1	human IAV	TRIG
Human-like H1N2	human IAV	human IAV	TRIG
H3N2	human IAV	human IAV	TRIG

##### 2.4.1.1. Classical H1N1 Virus

The first influenza virus known to have infected pigs is called the “classical” H1N1. It entered swine populations in 1918, concurrently with the highly virulent H1N1 Spanish flu epidemic in people [9,11,12,39]. During the 1918 human pandemic, the H1N1 human influenza virus was transmitted between people and pigs; outbreaks in farm families were often followed immediately by outbreaks in their swine herds, and outbreaks in pigs were sometimes followed by illness among humans on the farm [3]. While some authors suggest that the classical H1N1 virus might have entered swine populations from humans, the origins of this virus and direction of transfer are still unclear. The virus circulated in both humans and pigs after this time, but diverged significantly in the two host populations [41,42]. H1N1 viruses in people continued to change through antigenic drift. In contrast, classical H1N1 virus remained antigenically stable while it circulated in swine populations in North America [39].

##### 2.4.1.2. Triple Reassortant H3N2 Viruses and the Emergence of the TRIG Cassette

The classical H1N1 virus was the major IAV-S in North American pigs for 70 years [39]. Although some human H3 viruses were also found at low levels in pigs, no virus became established as a stable lineage during this time [39]. Triple reassortant H3N2 viruses were first detected in U.S. pigs in the late 1990s. They appeared first mainly in the Midwest [12,43,44,45], and they have been detected in Canada since 2005 [46,47,48]. These viruses contain the HA and NA proteins from human influenza viruses, and internal proteins from the classical IAV-S (NS, NP, M), avian influenza viruses (PB2, PA) and human influenza viruses (PB1) [39,44]. This particular combination of internal genes is known as the triple reassortant internal gene (TRIG) cassette.

The TRIG cassette seems to be particularly efficient in generating viruses with new HA and NA gene segments, and viruses carrying this cassette have reassorted with additional human influenza viruses, as well as with other influenza viruses in swine [39,49,50]. Many new viruses containing the TRIG cassette have been detected in North America since the triple reassortant H3N2 viruses emerged, and certain H3N2, H1N1 and H1N2 viruses have become endemic in swine populations. The H3 viruses arose from at least three separate introductions of H3 human influenza viruses into pigs [39]. HA of the H3N2 viruses in pigs can be divided into 4 distinct genetic clusters (I, II, III and IV) [39,51]; most of those circulating in recent years are in cluster IV.

##### 2.4.1.3. H1N1 and H1N2 Viruses with the TRIG Cassette

Reassortant H1N1 viruses (rH1N1), which contain the same neuraminidase and hemagglutinin as the classical H1N1 virus, but with the TRIG cassette, became common in North American pigs after 1998 [39,49,50,52]. H1N2 viruses with the TRIG cassette contain H1 from the classical H1N1 virus and human-origin N2 from the triple reassortant H3N2 viruses [39]. Since 2005, TRIG-containing H1N1 and H1N2 viruses with human-like H1, distinct from classical H1, have also become established in North American swine populations [39,50]. The N1 or N2 of these viruses is also of human lineage. Such “human-like” IAV-S viruses are often isolated from pigs with respiratory signs [39]. There are now four phylogenetic clusters of H1 viruses—alpha, beta, gamma and delta—endemic among pigs in the U.S., with variable cross-reactivity and cross protection between the clusters [50]. The δ cluster has been divided into δ1 and δ2, from what appears to be independent introductions of human seasonal H1 influenza viruses into pigs [53].

The N2 genes of current swine H1N2 and H3N2 viruses fall into two distinct lineages [54]. One of these (N2-1998) was introduced from human seasonal H3N2 during the original triple-reassortment event of the late 1990s. The other (N2-2002) was acquired from a human seasonal H3N2 virus that circulated around 2001–2002.

##### 2.4.1.4. 2009 Pandemic H1N1 and Its Reassortants

Many herds were also infected with the 2009 pandemic H1N1 virus circulating among people. This virus entered human populations in 2009, and spread throughout the world. It originated from North American and Eurasian IAV-S strains that underwent gene reassortment [55,56]. The HA of this virus appears to come from classical H1N1 IAV-S, and the NA from an avian-like H1N1 virus that circulates among pigs in Eurasia. The pandemic H1N1 virus has been introduced repeatedly into swine herds throughout the world [13,40,57,58,59]. This virus has undergone reassortment with IAV-S in the US, Canada, and other locations around the world [13,40,54,58,59,60,61,62]. The M gene and other internal genes from the pandemic virus have been found in many H1 and H3 IAV-S strains [54].

##### 2.4.1.5. Other Subtypes

Other variants of the dominant subtypes are also reported periodically among pigs (e.g., [43]), and additional subtypes have been found occasionally. H2N3 viruses were isolated from pigs on two farms in the central U.S. in 2006 [63]. The H2 and N3 in these viruses were from avian influenza viruses in waterfowl (possibly different bird species), and the PA was also of avian lineage, but they contained the other genes from the TRIG cassette [63,64]. This virus did not seem to affect other herds [64]. However, its H2 bound well to mammalian receptors, and it might be able to replicate readily in mammals [39]. H3N1 viruses have also been found [65,66], but were composed of swine-lineage H3 and N1 and thus, less of a risk to immune pig populations than truly novel subtypes such as the H2N3.

#### 2.4.2. Influenza A Viruses in Swine on Other Continents

With the globalization of world economies, there is a risk of introducing influenza viruses from other regions into the U.S. H3N2, H1N1 and H1N2 viruses circulate among pigs in Europe and Asia, and other subtypes such as H3N1 have been found [11,12,13,40,67,68]. Despite the superficial similarity in subtypes, many of these viruses are different from the viruses found in North America. For example, an H1N1 virus, which has all gene segments of avian origin, has circulated among swine in Europe since the 1980s [40]. H3N2 viruses in Europe have HA and NA of human influenza A virus origin, but do not contain the TRIG cassette [11,40]. Pandemic H1N1 has infected swine herds throughout the world, and in some cases, this virus has undergone reassortment with local IAV-S strains [13,40].There is currently little information about IAV-S subtypes in Mexico or South America where H3N2 and H1N1 viruses are known to circulate, but genetic characterization has rarely been reported [69]. The prevalence of IAV-S subtypes and lineages in African countries is also not well characterized [70].

## 3. Mechanisms of Immunity to Influenza A Viruses

Immunity to viruses is a complex process involving both nonspecific (innate) protective responses, and specific (adaptive) humoral and cell-mediated immunity directed against the specific virus. The various cells and soluble mediators of the immune system interact in many ways to eliminate the pathogen. The lungs present a particular challenge for immune responses, as viruses must be eliminated, but severe inflammation must also be avoided to minimize tissue damage.

### 3.1. Innate Responses to Influenza A Viruses

Innate responses include chemical, physical and cellular responses that are immediately protective against a broad range of invading microorganisms. These reactions are often triggered by the “recognition” of certain motifs that are found in microorganisms, but are not normally present in the host. For example, the viral RNA of influenza A viruses is recognized by pattern recognition molecules in infected cells, and triggers the production of proteins called type I interferons (interferon α and interferon β) [39,71]. Type I interferons, in turn, have a number of antiviral properties that inhibit influenza virus replication [39]. Conversely, the influenza A virus protein NS1 can inhibit interferon-mediated host defenses [24,25,26,72]. Other components of innate responses, including macrophages [73] and natural killer (NK) cells [74] also play roles during influenza A virus infections. Innate immune responses contribute to the activation of adaptive immunity [72].

### 3.2. Adaptive Immune Responses to Influenza A Viruses

Adaptive immune responses are traditionally divided into humoral immunity, which includes those mechanisms that are dependent on antibodies, and cell-mediated immunity (CMI), in which immune cells (cytotoxic T cells) directly recognize and destroy infected host cells. Antibody responses can be induced by any protein that enters the body, including those of killed viruses (e.g., in inactivated influenza vaccines for swine). Cytotoxic T cell responses are induced optimally by live organisms (such as IAV) replicating inside cells.

The critical immune cell types for both humoral immunity and CMI are various types of lymphocytes (B or T lymphocytes). Each lymphocyte responds very specifically to the immunogenic portions of that virus, called antigens. Most antigens are proteins. In reality, each lymphocyte actually responds to a tiny piece of the viral protein, called an epitope. Epitopes may be shared between strains of influenza viruses, or may be unique to a strain. Depending on the epitopes that are recognized, immune responses may be more or less broadly protective against different viral strains. Some subpopulations of responding lymphocytes also differentiate into memory cells, which respond more strongly when they encounter the same virus a second time.

#### 3.2.1. Adaptive Immunity: Humoral Response

##### 3.2.1.1. The Generation of Humoral Immune Responses to Influenza A Viruses

Antibodies are important in preventing influenza virus infections and in reducing the severity of disease [39]. Antibodies are made by B cells (also called B lymphocytes). B cells can make different isotypes of antibodies. They initially make IgM, then switch to producing other isotypes such as IgG or IgA. Antibody isotypes differ in their ability to carry out the various humoral immune responses that defend the body from pathogens. Helper T cells are critical for the production of high quality IgG and IgA antibodies that have high affinity to the antigen.

Antibodies to the HA of IAV-S can be found in the respiratory tract of pigs as soon as 4–5 days after infection with IAV-S [75,76], and serum titers appear in approximately 7 days [77,78,79], although peak levels occur later (e.g., 2 weeks [75]). Both local IgA and IgG are found in the respiratory tract of influenza virus-infected pigs [39,75,76,78,80,81]. Protective antibody responses in the lungs are often, but not always, correlated with the serum antibody titers in the blood [39,82,83]. Even though antibody titers measured in the blood fall gradually over time, influenza-specific B cells might be maintained throughout a pig’s life. In laboratory animals such as mice, memory B cells specific for influenza A viruses can be found for many months in the respiratory tract and other tissues [71]. Humans can develop long-term antibody responses to some antigens, possibly spanning many decades [71].

##### 3.2.1.2. Antibody Responses to the HA Protein

The influenza A virus HA usually induces the strongest antibody responses after infection [84], probably because this protein is present in large amounts on the surface of the virus. Antibody titers to the HA are, however, affected by the dose of virus [71], and possibly its subtype. In some studies, pigs infected with H3N2 viruses had higher serum antibody titers than pigs infected with H1N1 or H1N2 viruses [33,79,85].

Some antibodies to the HA, called neutralizing antibodies, can block this protein from attaching to its sialic acid receptor, and thus prevent the virus from infecting cells. Well-matched antibodies to the HA can be sufficient to prevent an influenza virus infection, and also contribute to clearing the virus from the lungs [71,86]. Whether the antibody response to the HA can protect the animal from a different viral strain depends on the similarity between the HA proteins of the two viruses [72]. This is influenced not only by the overall genetic similarity between these proteins, but on how well the individual epitopes match; changes in some parts of the HA can influence protection more than others [87]. Neutralizing antibody responses are thought to be the most important immune responses for protecting pigs against closely related IAV-S of the same subtype [71,80,83].Protective immunity mediated by neutralizing antibodies can be predicted by the magnitude of serum antibody activity against the particular IAV-S strain, e.g., by the serum hemagglutination inhibition (HI) titer [39] or by serum neutralization (SN) tests [88,89].

##### 3.2.1.3. Antibody Responses to the NA Protein

Animals exposed to influenza viruses normally make fewer antibodies to the NA than the HA [84]. Humoral responses to the NA have not been studied as extensively, but they may also be important in protection [28,29,30,31,72]. Although they do not prevent infections, antibodies to NA seem to contribute to reduced virus shedding and/or less severe illness [28,29,30,31,80,86]. Antibodies to the NA are thought to reduce the ability of the virus to spread from cell to cell, by preventing this enzyme from cleaving sialic acids [72,86]. They might also prevent the NA from clearing a path through the respiratory tract mucus layer and cell glycocalyx [86]. Similarly to the HA, mutations in the NA can reduce the protective effects of pre-existing antibodies [72], although the NA has a somewhat slower mutation rate [90,91,92].

##### 3.2.1.4. Other Roles for Antibody

Antibodies might also contribute to immunity against influenza viruses by alternative mechanisms. In some cases, antibodies can promote the ingestion of viruses by phagocytes (cells, such as macrophages, which engulf and destroy many pathogens). These cells have receptors that can recognize part of the antibody molecule, essentially tethering the virus to the cell via the antibody. The virus is then ingested and destroyed. Similarly, some immune cells, such as macrophages, can recognize antibodies attached to viral antigens on infected cells, and destroy the infected cell. This mechanism is known as antibody-dependent cell cytotoxicity (ADCC). Antibodies to both the viral HA and NA can mediate ADCC, and may contribute to clearance of influenza viruses [72].

#### 3.2.2. Adaptive Immunity: Cell-Mediated Response

Based on studies in mice, cell-mediated immune responses (CMI) are thought to be critical for complete viral clearance once the influenza virus has infected cells [93]. There is evidence that these responses also occur in the lungs of pigs infected with IAV-S [81,94,95]. However, CMI responses do not prevent infection [80]. In humans, cell-mediated immunity to influenza was correlated with reduced virus shedding, when antibodies to the virus were absent [96].

CMI is primarily mediated by cytotoxic T cells. These T lymphocytes play an important role in eliminating influenza virus-infected cells [73]. They recognize fragments of viral proteins (epitopes) displayed on the surfaces of infected cells in MHC I molecules. CMI responses are usually directed to the internal, conserved proteins of influenza viruses [71,80,97], especially the NP [98,99,100].Cytotoxic T cells eliminate influenza virus-infected cells through two major mechanisms [101]. In one, they produce molecules called perforins, which insert themselves into the cell membrane of the infected cell and lyse it. In the other, the T cytotoxic cell triggers cellular receptors that cause the infected cell to self-destruct (Fas/FasL mechanisms). In pigs, activated T cells have been reported as early as 5–7 days after infection with influenza viruses [77,95].

Protection between less closely related influenza viruses seems to depend, in part, on CMI reactions to the conserved internal proteins of the influenza virus [71,72,80,87,102,103,104,105]. It is not practical to measure CMI outside the research laboratory setting; however, it is important to realize that some critical immune responses to the virus may take place independently of antibody titers measured in the blood. In addition, inactivated influenza vaccines for swine stimulate limited CMI compared to natural exposure, limiting the breadth of protection they can provide.

#### 3.2.3. Immunity from Vaccination

Although other vaccine types are in development, most IAV-S vaccines licensed in the U.S. contain inactivated whole viruses of the H1 and H3 subtypes [38]. Factors that may influence vaccine efficacy include homology between the vaccine and challenge strains, the immunogenicity of vaccine components, the quantity of antigen included, and the adjuvant used. Matching of the viral HA is a critical component in vaccination because the goal of inactivated vaccines is mainly to produce neutralizing antibodies to this protein [80]. Responses to the NA may also be protective [28,29,30,31]. While there have been reports of CMI responses to inactivated influenza vaccines in swine [106], CMI and mucosal immune responses to these vaccines are generally thought to be very limited [94,107,108]. A poor CMI response to an inactivated vaccine limits its ability to cross-protect against strains with antigenically divergent HA and NA proteins. A recent study tested an intranasal IAV-S vaccine containing inactivated virus and a synthetic adjuvant called poly (I:C) [109]. Compared with a conventional inactivated vaccine, the intranasal poly (I:C) adjuvanted vaccine induced higher cross-reactive serum HI antibody titers and somewhat greater protection against heterologous challenge.

## 4. Maternal Antibodies to Influenza A Viruses in Swine

Newborn piglets have no maternal antibodies at birth, as these proteins cannot cross the placenta in swine [1]. Although neonatal piglets are able to mount immune responses, their immune system appears to be underdeveloped at birth, and less able to respond to pathogens and vaccines for the first few weeks [110]. Maternal IgA, IgG and IgM antibodies to IAV-S and other pathogens are transferred to piglets in colostrum from the dam, usually during the first 36 h of life [110]. Lymphocytes and other cells are also present in the colostrum, and can cross the intestinal lining in the neonate during this “open gut” period [110]. In addition, immunomodulatory factors received in colostrum might aid the development of the newborn’s own immune system [110]. Although the gut closes to the absorption of antibodies and cells after approximately 36 hours, piglets continue to receive passive mucosal IgA antibodies in their gastrointestinal tract from the milk until weaning [1].

A common strategy to control IAV-S is to vaccinate sows, which then transfer this protection to their piglets in colostrum. As the maternal antibodies decline, however, the piglets become susceptible to infection. In the field, where most sows have been infected with IAV-S, vaccination often results in significantly higher and relatively uniform HI titers (often ≥160) compared to unvaccinated sows [79]. In the offspring of these sows with high titers, maternal antibodies have been found to persist until 14–16 weeks, while they often disappear around 6 weeks in piglets born to exposed but unvaccinated dams ([111] cited in [79]).

### 4.1. Maternal Antibody Inhibition of Active Immune Responses in Piglets

Although they may protect the piglet, maternal antibodies can interfere with the development of active immunity after infection or vaccination [110]. In several studies, piglets with maternal antibodies had no rise in HI titers after they were exposed to IAV-S [112,113,114,115,116]. Other groups reported that such piglets did respond to infection or vaccination, though more weakly. In one study, 7-week-old piglets with low levels of maternal antibodies developed lower HI titers, virus-specific immunoglobulins, and T cell proliferative responses compared to piglets without maternal antibodies, when they were infected with the same H1N1 virus that had been used to vaccinate their dams [117]. Nevertheless, these pigs did mount an immune response that resulted in less severe clinical signs, compared to naive pigs, when they were re-exposed to the virus at 15 weeks of age. In another study, piglets vaccinated with a bivalent inactivated vaccine at 1 and 4 weeks, 1 and 8 weeks, 4 and 8 weeks, 8 and 10 weeks, or 8 and 12 weeks of age had detectable HI titers after the second dose [118]. The titers were lower in piglets with passive maternal antibodies. Other studies have shown that it is possible for piglets to develop cell-mediated immune responses (CMI) to antigens despite the presence of maternal antibodies [110]. Results of another study suggest that inactivated IAV-S can induce greater HI antibody titers and cross-protection in maternal antibody-positive piglets if it is formulated with poly (I:C) adjuvant and administered intranasally [109].

### 4.2. Maternal Antibody-Mediated Protection from Disease

Maternal antibodies sometimes provide pigs with partial or complete protection from disease after inoculation with IAV-S [112,113,116,117], although protection may vary depending on the antibody titer [112]. One of these studies examined overall growth rates, as well as clinical signs, after two exposures to the same influenza virus [117]. In this study, 7-week-old piglets without maternal antibodies grew more slowly immediately after they were inoculated with IAV-S, compared to piglets with maternal antibodies; however, the animals in the two groups were similar in size 3 weeks later. When both groups of piglets were exposed to this virus again at 15 weeks of age, the piglets without maternal antibodies seemed to have higher growth rates overall. The explanation appeared to be that these animals were less affected by the second exposure to the virus. When piglets’ IAV-S specific maternal antibodies are poorly matched to a strain that subsequently infects them, there may be a risk of vaccine-associated enhanced respiratory disease (VAERD), a phenomenon described in Section 9.

### 4.3. Maternal Antibody Protection from Infection and Transmission of Virus

Piglets protected from clinical signs by maternal antibodies may still become infected and shed virus [114,116,117,119]. In some experiments, piglets with low levels of maternal antibodies shed IAV-S at least as long [112] or longer [117] than piglets without maternal antibodies. In contrast, high maternal antibody titers inhibited virus shedding in one of these two studies [112]. Neonatal piglets that become infected might act as reservoirs for influenza viruses under some field conditions even when viruses are not detected in the breeding sows [120,121]. An H1N1 virus could be detected for up to 70 days in one group of growing pigs housed in a finishing barn, when no additional animals were added to the group [120]. Piglets with waning maternal antibodies could be infected with IAV-S from older pigs or other sources of the virus, and these viruses may be transmitted relatively slowly among the growing pigs. A small number of animals infected at weaning might act as the source of virus for the rest of the cohort, and could also transport the virus to distant production units. Vaccination of sows might be able to decrease virus transmission in some cases. In one study, high and uniform titers of maternal antibodies to an H1N1 virus reduced the transmission of the same virus, although it did not prevent the transmission of an H1N1 virus that belonged to a different phylogenetic cluster [122]. Supportive evidence is also provided by a report from the field, in a herd where mass vaccination of sows was able to boost maternal antibody levels sufficiently to stop an outbreak of respiratory disease among suckling and nursery piglets [7]. Based on these studies, sow vaccination often provides piglets a measure of protection, but stopping virus transmission in the herd requires a close antigenic match between the sow vaccine and the local circulating strain(s).

## 5. Influenza Surveillance in Swine

New subtypes or strains of influenza resulting from antigenic shift or drift may produce an increased threat to animal and human health. These emerging influenza variants can be identified by monitoring changes in circulating influenza virus strains in swine. The integrated and coordinated USDA Swine Influenza Virus Surveillance System strategy was developed due to concerns about antigenic drift and gene reassortment of IAV-S strains, as well as sporadic reports of human infections with IAV-S. Program details are available at USDA’s Swine Influenza Surveillance website [123]. Documents included at this website are as follows:

Influenza Surveillance in Swine Procedures Manual [124]:

Appendix A: National Surveillance Plan for Swine Influenza Virus in Pigs [125]

Appendix B: AVIC and State Veterinarian Directory

Appendix C: Testing Guidelines, Forms, Submission Instructions

Appendix D: Designated NAHLN SIV Testing Laboratories

Appendix E: Notification Plan

Appendix F: SIV Specimen Submission Form for Regulatory Veterinarians

Quick Reference Guide on USDA Swine Influenza Virus (SIV) Surveillance

Influenza Virus Surveillance in Swine - Program Overview for Veterinarians (color brochure)

Producer Guide to Influenza Virus Surveillance in Pigs (color brochure)

### 5.1. Program Objectives

The surveillance program was designed by the USDA Animal and Plant Health Inspection Service (APHIS), the USDA Animal Research Service (ARS), industry stakeholders and public health officials with the intent to foster robust scientific research that would address veterinary and public health concerns [123,125]. Implementation of the program formally began in 2010. The objectives of this surveillance program are to:

1. Monitor genetic evolution of endemic influenza in swine to better understand endemic and emerging influenza virus ecology;
2. Make available influenza isolates for research and to establish an objective database for genetic analysis of these isolates and related information; and
3. Select proper isolates for the development of relevant diagnostic reagents, updated diagnostic assays, and vaccine seed stock products.

In designing the program to meet these objectives, special care was taken to give veterinarians and producers flexibility to participate without concern that IAV-S detection would bring scrutiny to their operations. Sharing of data and viral isolates is encouraged so that public health authorities, researchers and manufacturers will be equipped with information and materials that are as contemporary as possible. To meet both of these goals, veterinarians and producers are given the option to submit specimens anonymously.

### 5.2. Targeted Swine Populations

In order to optimize resources and provide a level of uniformity in sampling, the surveillance program is focused toward sampling from swine that meet specific criteria. The following swine populations are targeted for the IAV-S Surveillance Program [123]:

1. Case-compatible swine accessions submitted to veterinary diagnostic laboratories. This stream includes samples submitted by producers, veterinarians or other personnel who observe pigs exhibiting influenza-like illness (ILI) on farms. Samples collected for routine diagnostic testing may also be tested at the laboratory for influenza as part of the surveillance program.
2. Swine exhibiting ILI at first points of concentration or commingling events such as auctions, markets, fairs or other swine exhibition events. Animal health officials or licensed veterinarians may observe pigs with ILI at events with an increased potential for influenza transmission and/or elevated human exposure. The veterinarian in charge or animal health official may segregate, examine and collect appropriate samples from pigs exhibiting ILI. These samples would be submitted to a veterinary diagnostic lab and tested for IAV-S as part of the surveillance program.
3. Swine populations epidemiologically linked to a confirmed isolation of IAV-Sin a human. When a person tests positive for influenza that may be linked to swine exposure, that individual’s identity is known to public health officials and possible sources for infection will be known to them. The U.S. influenza surveillance system for humans collects influenza activity information submitted voluntarily by public health partners and health-care providers. The surveillance information is divided into five categories including virological surveillance, outpatient illness surveillance, mortality surveillance, hospitalization surveillance and summary of the geographic spread of influenza [126]. Occasionally, an epidemiological link may be suspected when human influenza cases have a recent history of exposure to pigs. Samples are collected in cooperation with the owner/producer and herd veterinarian from swine that are epidemiologically linked with a human influenza A case. The number of samples collected will be determined on a case-by-case basis.

### 5.3. Sample Collection

Samples may be collected and submitted from producers, veterinarians, animal health officials or other personnel [123]. Animals to be sampled should be febrile if possible (some IAV-S strains induce a minimal febrile response) with serous nasal discharge and cough. Samples may include nasal swabs, oral fluids, and lung tissue. Up to ten samples from animals which fit the case definition may be sampled per diagnostic case under the IAV-S Surveillance Program. Lung tissue collected from mortalities or euthanized animals may be submitted in plastic bags or screw-top plastic tubes. When collecting samples using nasal swabs, polyester or flocked swabs are inserted deep into the nasal cavity while the pig is restrained. The swab is rotated to collect surface epithelium as well as mucosal secretions from both nostrils. Blood on the swabs may interfere with testing, so caution should be taken in rotating too vigorously or inserting the swab too deeply. Swabs should be inserted into virus transport media. Once the samples are collected, they should be chilled until shipped or shipped as soon as possible with ice packs.

### 5.4. Testing, Reporting and Response

Testing for the IAV-S Surveillance Program is performed through the National Animal Health Laboratory Network (NAHLN) which is composed of state or university laboratories [123,124]. The network is organized and supported so that it has the capacity to respond to animal-disease outbreaks nationwide and is the model for effective diagnostic networks, able to respond and communicate diagnostic outcomes to decision makers quickly. NAHLN laboratories perform routine diagnostic tests for endemic animal diseases (like IAV-S) as well as targeted surveillance and response testing for foreign animal diseases in the event of an outbreak. NAHLN laboratories that conduct the testing for the IAV-S Surveillance Program are listed online [123]. Samples are tested from the following swine populations.

#### 5.4.1. Case-Compatible Swine Accessions

As with any routine diagnostic work-up, NAHLN diagnostic labs conduct tests requested by the veterinarian and report test results to the submitting veterinarian as per the usual process. In addition to the routine testing, NAHLN labs may conduct additional testing of samples for the IAV-S Surveillance Program [123,124].

IAV-S Surveillance Program test results are reported as anonymous unless the producer is willing to participate in the traceable surveillance option [123,124]. Producers participating in the traceable surveillance option receive surveillance test results. No additional charges to the submitter result from testing conducted in accordance with either the anonymous or traceable surveillance streams of the IAV-S Surveillance Program.

Data are collected by NAHLN, or through direct messaging between laboratory information management systems. Data are then monitored and analyzed by the USDA, in collaboration with industry stakeholders and the office of the State Animal Health Official (SAHO), to identify sequences and data that may initiate further research or targeted surveillance. Data confidentiality and security are priorities. When submitted through traceable surveillance, the response to unusual test results is determined on a case-by-case basis.

#### 5.4.2. Targeted Surveillance of Sick Pigs at First Points of Concentration or Commingling Events

If a SAHO determines samples should be collected, the attending veterinarian and/or regulatory officials collect and submit samples from these animals to a NAHLN lab for IAV-S testing. As with the case-compatible swine accessions, unless written permission is provided by the owners of pigs sampled for traceable surveillance, test results will be entered through the anonymous surveillance stream. The SAHO determines the control measures, if any, which will be utilized at the event prior to or after receiving test results.

#### 5.4.3. Surveillance of Swine Populations Epidemiologically Linked to a Human Case of IAV-S

With producer permission and under the direction of the SAHO, personnel collect samples from swine exhibiting ILI. Samples are submitted to the National Veterinary Services Laboratories (NVSL) through traceable surveillance. Under the herd’s licensed veterinarian’s supervision, movement of the animals occurs when clinical signs have resolved to satisfactory levels.

### 5.5. Benefits of the IAV-S Surveillance Program

Perceived and real risks to the health of pigs and people from influenza infection fuels the need for the information gained from the surveillance efforts. Testing field samples submitted under the IAV-S Surveillance Program has several immediate and long term benefits to both producers and the public. In the short-term, the information is used to make decisions addressing disease control and prevention measures, human health concerns and trade issues. In addition, researchers and the animal health industry can use the information to develop and update relevant diagnostic reagents, targeted influenza diagnostic assays, effective vaccines and response plans.

During FY2013 (October 2012-September 2013), 21725 pigs were tested through diagnostic laboratory submissions from 4991 accessions. Of the 1579 Influenza A positive accessions, the accessions with each subtype tested as follows: 519 H1N1, 380 H1N2 and 286 H3N2. Fifty-four accessions contained mixed subtypes [127]. An increase in swine diagnostic lab submissions through the IAV-S Surveillance Program will continue to provide valuable information to the industry, researchers and public health officials. Sequenced virus information from samples submitted through the IAV-S Surveillance Program is available to researchers via the public database GenBank^®^ (http://www.ncbi.nlm.nih.gov/genbank/). IAV-S isolates in the NVSL repository are available to researchers and to firms developing vaccines and diagnostic kits.

Over the long-term, the information may be used to help understand influenza infection in pigs, to gain a better understanding of epidemiological factors affecting the mutation and spread of IAV-S in the swine population, and to provide information for broader initiatives such as Veterinary Services 2015 Project, Surveillance for Action, a stream-based comprehensive and integrated animal disease surveillance system.

While the IAV-S Surveillance Program was initiated in 2010 in the U.S. with a focus on monitoring the pandemic H1N1 2009 (H1N1pdm09), IAV-S surveillance has also been occurring in other areas of the world. Internationally from 2009–2011, introductions of the H1N1pdm09 influenza virus from humans into swine were observed in 12 countries and regions. Due to the high level of surveillance and transparency, a majority of reported introductions occurred in the U.S. and Canada (19 and 8, respectively) [128]. Transmission of seasonal influenza viruses from humans to swine is also being monitored in the U.S. and globally. From 1990 to 2011, at least 23 separate introductions were documented, with six first identified in the U.S. and three in Canada [128].

Monitoring the transmission of influenza viruses from humans to pigs aids in understanding the contribution of human-origin influenza viruses to the genetic diversity of IAV-S. There have been several instances in the past 15 years when gene reassortment between human seasonal and swine viruses produced new IAV-S lineages that became established in the swine population [54]. When virus transmission increases between humans and pigs, measures to prevent transmission need to be followed. Increased biosecurity measures and utilization of influenza vaccination for swine workers and pigs may be necessary to lower the risk of reassortment between human and swine viruses.

A surveillance program is most useful (e.g., for selection of well-matched vaccine strains) if participation is high and sampling bias is minimal. The IAV-S Surveillance Program currently focuses on testing specimens submitted from swine herds affected by respiratory disease. If some regions are under-reported or over-reported it may skew the estimation of strain frequencies, which could affect the selection of widely representative vaccine strains. Complementary data could be gained through a sentinel surveillance component, which involves sampling representative herds at regular intervals, independently of disease incidence. Sentinel surveillance could therefore help to identify a full cross-section of circulating IAV-S strains.

## 6. Tools for Characterizing Field Strains of IAV-S

Many different diagnostic tools are available to laboratories for the detection of influenza infection in pigs. Samples collected by producers and veterinarians are tested with pen side tests or submitted to NAHLN or private laboratories. Which test is conducted depends on the sample submitted and the tests available at that specific laboratory.

### 6.1. Samples Submitted

Various samples including serum, lung tissue, nasal swabs, tissue swabs and oral fluid are being collected in the field for influenza testing. Snout wipes have recently been introduced and are still very new in the diagnostic world. Details of each sample collection are included below.

#### 6.1.1. Serum

Serum collected at least one week after the start of infection may contain antibody. One week after infection, pigs may have titers of at least 80. These titers can increase to 320 to 640 when sampled 14–21 days after infection [129]. Paired samples may be especially useful in vaccinated herds.

#### 6.1.2. Lung Tissue

Lung tissue is collected from mortalities or euthanized animals and placed into Whirl-Pak^®^ or screw top-plastic tubes. Tissue from each animal sampled should remain in an individual bag for submission.

#### 6.1.3. Nasal Swabs

When collecting samples using nasal swabs, polyester or flocked swabs are inserted into the nasal cavity while the pig is restrained. The swab is rotated to collect surface epithelium as well as mucosal secretions from both nostrils. However, if swabbed too aggressively, blood may interfere with IAV-S testing [130] so caution should be taken in rotating too vigorously or inserting the swab too deeply. The swab is placed in a tube containing medium or sterile saline or alternatively, the ampule at the end of the sheath is squeezed and medium is released onto the swab (if applicable).

#### 6.1.4. Tissue Swabs

Polyester or flocked swabs are inserted into small airways in lung tissue taken from post-mortem necropsy [131]. The swab is rotated to collect surface epithelium. The swab is placed in the tube with medium or sterile saline or alternatively, the ampule at the end of the sheath is squeezed and medium is released onto the swab (if applicable).

#### 6.1.5. Oral Fluid

Oral fluid samples can be collected on an individual animal basis, or alternatively on a group basis by hanging ropes in pens [132]. Cotton ropes are suspended over the pen, at shoulder height for the pig and away from feed and water. After 20–30 min, the oral fluid is extracted from the rope either by placing a plastic bag over the hanging rope and stripping the fluid from the rope into the bag or by cutting off the rope, placing it into the bag and squeezing out the fluid. A corner of the plastic bag can be cut to allow the fluid to be collected into a plastic tube.

#### 6.1.6. Snout Wipes (Not Currently Accepted in the USDA Surveillance Program)

One case study compared diagnostic results from testing nasal swabs *versus* snout wiping [133]. Snout wiping is performed by using a disposable household cleaning pad soaked in saline. The pad is rubbed over a piglet’s nose then placed in a sealed plastic bag (up to five pigs’ noses may be rubbed with one pad as a way to pool samples). A corner of the bag is cut and the pad is squeezed so the liquid runs into a plastic snap tube for submission to the laboratory. In this case study, virus isolation and sequencing were successful, and the sequencing information was utilized to help select a vaccine for breeding animals. Although this sample collection technique shows great promise, validation studies still need to be performed before snout wipes become a widely recommended sampling technique.

### 6.2. Diagnostic Testing

Accurate, cost effective IAV-S diagnostic testing with a rapid turnaround time is desired by veterinarians in the field who are making recommendations for IAV-S control or prevention in swine herds. Reliable diagnostics provide critical information to assist veterinarians in the decision making process. Several diagnostic tests are available, but laboratories vary in the tests they offer to clients.

#### 6.2.1. Antibody Testing

##### 6.2.1.1. Hemagglutination Inhibition Test

According to the OIE Manual of Diagnostic Tests and Vaccines for Terrestrial Animals 2013, the hemagglutination inhibition (HI) test is the main serological test performed to detect IAV-S antibodies [134]. Serum HI antibodies are also considered the gold-standard correlate of protection from inactivated IAV vaccines. This test is conducted by adding serial dilutions of the submitted serum samples to a known concentration of virus. A titer is determined by the degree to which antibodies in the serum samples bind the virus in the test plates, thus preventing agglutination of the indicator erythrocytes. Paired serum samples collected 10–21 days apart are ideal. A titer increase of four-fold or greater between the two samples suggests a IAV-S infection [129]. The HI test is easy and quick to perform [129]. However, the success of this test depends on whether the virus strain used in the test and the field strain are antigenically similar, so laboratories may need to test samples against a panel of IAV-S strains [135].

##### 6.2.1.2. Enzyme-Linked Immunosorbent Assays

Lee *et al.* developed a subtype specific indirect ELISA to detect HA-binding antibodies from swine exposed to H1N1 or H3N2 influenza viruses [136]. Commercial test kits for ELISA-based subtyping were available [137,138], but are no longer sold in the United States. Another commercial ELISA kit detects antibodies to the more conserved nucleoprotein (NP) of diverse IAV-S and avian influenza strains, which provides a broad-spectrum screening test [139]. This indirect ELISA assay against the NP protein has gained in use in recent years due to the complexity of antigens needed for HI assays and its flexibility to test sera from multiple species.

##### 6.2.1.3. Additional Serological Tests

Other serological tests developed but not commonly used include virus neutralization, agar gel immunodiffusion, and indirect fluorescent antibody assays [134].

#### 6.2.2. Influenza Virus Identification

##### 6.2.2.1. Culture

(1) Cell Culture Virus Isolation

Influenza virus can be isolated through cell culture from lung tissue and nasal swabs [134]. MDCK cells or primary porcine kidney cells can be utilized. This test may take 2–3 days to perform, which is longer than many virus detection methods [129]. It is more commonly used to characterize the virus and to isolate the virus when producing autogenous vaccine rather than for routine diagnosis [129,135]. Although this test is not offered by all laboratories, it is a requirement for fulfillment of the USDA IAV-S testing algorithm.

There is a short window of opportunity for IAV-S isolation from infected pigs, so isolation attempts often fail. Therefore it is important to select specific animals that are most likely to be shedding virus. It is best to identify pigs with fever and other clinical signs [140], but some strains of IAV-S may not cause acute disease. Selecting pigs based on a qualitative rapid detection kit (e.g., Flu DETECT^®^, Synbiotics Corp., Kansas City, MO, USA) is likely to improve the odds of successful virus isolation.

(2) Egg inoculation

Egg inoculation can be performed on lung tissue and nasal swabs and has been considered one of the better methods for influenza testing due to its sensitivity [141]. However, Swenson *et al.* determined that even egg inoculation is not 100% accurate [130]. Not all laboratories offer this test as it can be expensive to maintain the supply of embryonated eggs. Another drawback is that it takes multiple days to perform as with cell culture [129]. This test is utilized to isolate the virus when producing autogenous vaccine [135].

(3) Hemagglutination test

The hemagglutination assay is a relatively quick assay which determines if influenza virus is present. As a stand-alone test it is not highly sensitive. However, after specimen is passaged in eggs or cell culture, hemagglutination is a rapid method to detect amplified virus.

##### 6.2.2.2. Reverse Transcriptase PCR

Reverse transcriptase polymerase chain reaction (RT-PCR) tests may be performed on lung tissue, nasal swabs and oral fluid for positive/negative discrimination or for differentiating between H1N1 and H3N2 viruses [123,129]. Real-time RT-PCR (RRT-PCR) assays based on the influenza virus matrix gene have the ability to detect all subtypes of IAV [142,143]. A real-time RT-PCR assay on nasal swab samples was determined to be highly specific at 100% with sensitivity ranging from 88% to 100% [144]. PCR can be performed on oral fluid samples for virus detection, although several substances can interfere with virus isolation [135]. Assay development continues on oral fluid samples as they are an efficient approach to testing a large number of animals. Overall, RT-PCR tests can be performed more quickly than cell culture or egg culture isolation of the virus, but can be more expensive.

##### 6.2.2.3. Fluorescent Antibody Test

Fluorescent antibody (FA) testing, which detects IAV-S antigens and is performed on frozen sections of lung tissue, can be completed in hours. Both H1N1 and H3N2 can be detected and subtyped by FA [129]. However, the FA test does have its limitations. IAV-S antigens are only present in lung tissue for a short time following infection. In addition, autolytic changes in lung tissue will affect the test, so fresh lung tissue needs to be submitted [145].

##### 6.2.2.4. Immunohistochemistry Test

Immunohistochemistry (IHC) is an inexpensive, rapid and easy to perform test utilized to detect H1N1 and H3N2 influenza virus antigens on slides from formalin fixed tissue or nasal swabs [145]. IHC testing has been shown to have sensitivity equivalent to virus isolation and greater than FA [145]. As with FA, IAV-S antigens are only present in the lung tissue for a short time following infection, which limits the ability to detect the infection using IHC.

##### 6.2.2.5. Antigen-Capture Enzyme-Linked Immunosorbent Assays

Antigen-capture enzyme-linked immunosorbent assays (ELISAs) are commercially available to detect influenza in nasal swabs and lung tissue [130]. However, excess blood and mucus on nasal swabs or freezing lung tissue may reduce the sensitivity of the test [129,130]. Tissue swabs of airways have also been tested successfully [129,131].

##### 6.2.2.6. Typing Isolates

Isolates can be typed using hemagglutination and/or neuraminidase inhibition tests. These tests require a panel of reference antisera specific to each HA or NA antigen of interest. The hemagglutination inhibition test, outlined in the OIE Manual of Diagnostic Tests and Vaccines for Terrestrial Animals 2013, is more commonly performed because it identifies a clinical isolate’s HA serotype, which is usually of greater concern. The neuraminidase inhibition test is more cumbersome and likely to be performed mainly by reference laboratories [135].

##### 6.2.2.7. Sequencing

Complete or partial gene sequencing is often utilized to determine the subtype and genetic similarity to reference strains from the herd or to vaccine strains. Phylogenetic relationships of IAV genes may also be determined with these sequences and used to monitor virus evolution.

### 6.3. Testing for the IAV-S Surveillance Program

Samples collected from pigs under the case-compatible swine accessions or through targeted surveillance of sick pigs at first points of concentration or commingling events are tested at NAHLN laboratories while all samples associated with a human case are tested at the National Veterinary Services Laboratories (NVSL) in Ames, Iowa [123]. NVSL is the confirmatory laboratory.

NAHLN laboratories use the NAHLN assays for samples submitted into the IAV-S surveillance stream [123]. Matrix PCR is performed on submitted samples as a screening test. A positive matrix PCR indicates influenza nucleic acid is present. If the matrix PCR is positive, diagnosticians perform subtyping PCR and virus isolation to determine which influenza virus is present. For subtyping PCR, testing will determine whether the HA type is H1 or H3 and whether the NA type is N1 or N2. If the subtype is undetermined (for example as when a H5 isolate is being analyzed), additional testing will be utilized to subtype the virus. Virus isolation may be followed by gene sequencing. When sequencing is performed, hemagglutinin, neuraminidase and matrix genes are sequenced. Two ways to determine if the pandemic matrix is present are to sequence the M gene or use the differential/pandemic matrix PCR test that is available at NVSL and some NAHLN laboratories [123].

Matrix PCR, subtyping PCR, virus isolation and sequencing are tests available to the NAHLN laboratories. However, not all the NAHLN laboratories perform all four tests for IAV-S surveillance. The influenza test(s) each NAHLN laboratory is approved to perform are indicated online [123].

## 7. Best Use of Current Commercial Vaccines for IAV-S Control

### 7.1. Inactivated Influenza Virus Vaccines and Antigenic Matching

All of the licensed, conventional IAV-S vaccines in the United States (Table 3) contain inactivated (killed) virus. Each strain of virus is cultivated in eggs or cell culture and inactivated with chemical agents, such as formaldehyde or binary ethylenimine [134]. Inactivated viruses of multiple strains are blended and formulated with an adjuvant. Regulatory approval of an inactivated virus vaccine requires successful demonstration of safety and efficacy [134]. Efficacy is determined by experimental challenge of seronegative animals with the strain of virus identical to each vaccine seed virus, and sometimes with a heterologous strain. Since IAV-S strains are antigenically diverse and evolving, and many young pigs have maternal antibodies that can interfere with vaccines, the field efficacy of an approved product cannot be taken for granted. There are commercial vaccines that combine IAV-S antigens with antigens for other agents, such as *Erysipelothrix rhusiopathiae*, *Mycoplasma hyopneumoniae*, *Leptospira* species, or porcine parvovirus. Manufacturers must demonstrate that antigens in a combination product do not interfere with each other in terms of efficacy [146].

Conventional inactivated vaccines are effective in inducing protective immunity against antigenically identical or very similar strains [82,147]. In this ideal situation, the vaccine elicits systemic antibodies that can neutralize the virus very early without triggering extensive inflammation. If commercial killed IAV-S vaccines consistently included the predominant circulating North American strains, they would likely be more effective at preventing IAV-S outbreaks. The annual strain selection process for human seasonal influenza vaccines aims for this ideal. The human seasonal vaccines are reformulated most years, based on the results of global surveillance and identification of new strains that emerged through antigenic drift or shift [148,149]. This updating process, while imperfect, keeps the human inactivated virus vaccines approximately up-to-date with the evolution of A/H1N1, A/H3N2, and B viruses, so that vaccinated persons are more likely to develop antibodies that can neutralize the predominant circulating strains. A similar system of strain updating to support IAV-S vaccines for the United States has been advocated [150].

For IAV-S, maintaining close antigenic matches in the commercial inactivated virus vaccines is more difficult. First, the evolution of swine H1 and H3 viruses in North America has resulted in co-circulating strains that belong to about seven antigenically distinct clusters within the two subtypes (see Section 2.4) [51]. Second, IAV-S surveillance to monitor the evolution of strains within the United States is still a young program, and the geographical distribution of IAV-S genotypes across North America is far from homogenous [151]. Third, manufacturers of IAV-S vaccines make independent decisions about the strains contained in their products, and typically do not share those strains or publish their HA gene sequences.

**Table 3:** Licensed influenza vaccines for swine in the United States.

Vaccine Name	Manufacturer	IAV-S Lineages Included	Specific Strain Names (If Available)	Adjuvant	Combination Products
FluSure XP^®^	Zoetis	Delta-1 H1N2 Delta-2 H1N1 Gamma H1N1 Cluster IV H3N2	A/Sw/Oklahoma/0726H/2008 (H1N2) A/Sw/North Carolina/031/2005 (H1N1) A/Sw/Iowa/110600/2000 (H1N1) A/Sw/Missouri/069/2005 (H3N2)	Amphigen^®^	FluSure XP/RespiSure^®^ FluSure XP/FarrowSure GOLD^®^ FluSure XP/FarrowSure GOLD B^®^ FluSure XP/RespiSureONE^®^ FluSure XP/RespirSureONE/ER Bac Plus^®^
FluSure^®^ Pandemic	Zoetis	Pandemic H1N1	A/California/04/2009 (H1N1)	Amphigen^®^	
MaxiVac Excell^®^ 5.0	Merck Animal Health	Beta H1N1 Delta-2 H1N1 Gamma H1N1 Cluster I H3N2 Cluster IV H3N2		EMUNADE^®^	
PneumoStar^®^ SIV	Novartis Animal Health	Alpha H1N1 Cluster I H3N2		ImmunSTAR^®^	
Swine Influenza Vaccine, RNA	Harrisvaccines	Cluster IV H3N2		none	

Until 2007, updating strains in an IAV-S vaccine required a new licensure process for the product, including efficacy and field safety testing, which was recognized as a barrier to manufacturers making timely strain changes. In 2007, USDA-CVB introduced a new policy to allow manufacturers flexibility to change strains under an existing license, by either adding or replacing viruses, and demonstrating immunogenicity equivalent to that of the prior strains [152]. Despite this move to foster better matching between vaccines and the evolving IAV-S subtypes, strain updates over the following years have not been common. Reasons for this might include lack of confidence by firms that existing IAV-S surveillance data can identify better strains, and the time and expense required for regulatory approval, even under the 2007 revised policy. Recent serological data strongly suggest that antigenic drift among the Cluster IV H3N2 viruses has been substantial enough to reduce efficacy of the commercial vaccines [153]. With the strengthening of North American IAV-S surveillance in recent years [51,57], data are available to support more frequent and collaborative vaccine strain decisions.

### 7.2. Polyvalent IAV-S Vaccines Containing Multiple H1 and H3 Clusters

Several past and present IAV-S vaccines marketed in the US contained two strains of the H1 and H3 subtypes (End-FLUence2, Intervet Inc., Millsboro, DE, USA; FluSure, Pfizer Inc., New York, NY, USA; PneumoSTAR, Novartis Animal Health, Greensboro, NC, USA). Two of these were replaced several years ago with new products containing 4–5 strains of IAV-S (MaxiVac Excell 5.0, Merck Animal Health, Summit, NJ, USA; FluSure XP, Zoetis, Florham Park, NJ, USA) (Table 3). The greater valency of the newer products was a response to the emergence of antigenically distinct clusters within the H1 and H3 subtypes. This was an important development. Three of the earlier commercial vaccines that contained only a cluster I H3N2 strain were insufficient to reduce shedding of a cluster III H3N2 virus after challenge infection, whereas an experimental vaccine containing a cluster III virus conferred full protection [154]. A more recent experimental challenge study gave similar evidence that polyvalent commercial vaccines containing cluster IV H3N2 can protect against a drifted contemporary cluster IV strain better than a commercial vaccine containing only cluster I H3N2 [155]. Although antigenic drift is likely diminishing the quality of protection from the newer polyvalent vaccines [153,156,157], it is reasonable to assume these will protect herds more reliably than bivalent vaccines that contain even older IAV-S strains.

### 7.3. Field Strain Evaluation to Inform Choice of Current IAV-S Vaccines

**Figure 4:**
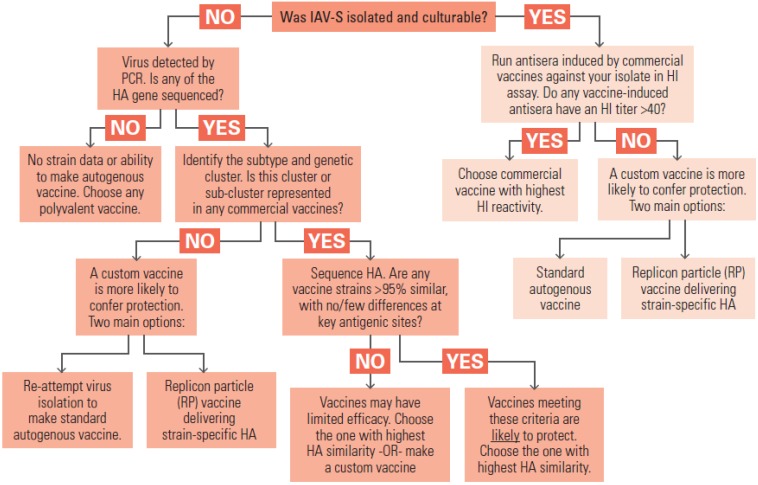
Decision tree for selection of IAV-S vaccine strategy to control a specific herd isolate. Due to the diversity and rapid evolution of IAV-S, there are currently no one-size-fits-all vaccine options. Since commercial polyvalent vaccines from different manufacturers contain different strains, diagnostic data (HA sequence and/or serological comparisons) may identify one vaccine that matches a specific field strain better than others. In some cases, data may indicate that commercial polyvalent vaccines offer no close matches to the field strain, suggesting a greater advantage for custom vaccines.

It would be beneficial if practitioners had more tools at their disposal to support a best-possible match between endemic IAV-S strains and the existing commercial vaccines. When field isolates are characterized, such as by serology or HA gene sequence, it would be valuable to relate their properties directly with the strains contained in available vaccines (Figure 4). If full-length HA sequences of all vaccine strains were available, one could identify which of those vaccines offers the nearest sequence homology to the field isolate of concern. Presently, such a service is available through the University of Minnesota Veterinary Diagnostic Laboratory. Major manufacturers of IAV-S vaccines privately share the HA sequences of their proprietary seed viruses with the laboratory. Diagnosticians can then compare HA sequence of a client’s field isolate against the vaccine seed viruses, quantifying the genetic similarity of all of them and identifying the vaccine most likely to provide an antigenic match.

A similar approach was reported in a case study by Corzo *et al.* [7], in which a large swine breeding herd was affected by a persistent outbreak of acute respiratory disease in piglets. Virus isolated from suckling piglets was sequenced, revealing a δ-1 cluster HA. Knowing HA nucleotide sequences of strains in the quadrivalent Flu-Sure XP^®^ vaccine (Zoetis, Florham Park, NJ, USA), the study team determined that the δ-1 cluster HA gene in the vaccine was nearly 99% homologous to the field isolate. This provided reasonable assurance that Flu-Sure XP contained HA antigen well-matched to the local circulating virus, and after mass vaccination of all breeding females the outbreak came to an end. However, sequence homology will not necessarily predict cross-protection because some sites in HA influence antigenic properties more than others. In a recent study, six critical amino acid locations were identified in H3; substitutions at one or two of those positions were sometimes enough to markedly change cross reactivity in the HI assay [158].This suggests that HI or neutralization assays should be conducted in addition to sequence analysis.

One relevant way to evaluate the match between field strains and vaccine viruses is to test vaccine-induced antisera for reactivity against field isolates, using HI or neutralization assays. This addresses the question, “Which of the available vaccines actually induces antibodies that most effectively block replication of my virus isolate?” Major manufacturers already provide some veterinary diagnostic laboratories (e.g., University of Minnesota and Iowa State University) with swine antisera raised against the individual IAV-S strains contained in their polyvalent products. This enables the diagnostic laboratories to compare the serum antibodies induced by available vaccines in terms of activity against IAV-S field isolates. It is roughly parallel to the idea of antibiotic sensitivity testing, where a laboratory analyzes bacterial field isolates for inhibition by the available antibiotics, reporting the most favorable options back to the practitioner.

After conversations with diagnostic laboratories and manufacturers, we suggest that this approach could be adapted further by using swine antisera induced by the licensed polyvalent vaccines (instead of monovalent components of the vaccine), administered in accordance with the approved product labels. First, this would be more efficient because it only requires one test for each of the available vaccines, instead of several tests to look at monovalent components one-by-one. Second, the results might be more realistic because any interactions between the multiple strains in the vaccine (positive or negative) would be accounted for. Similar strategies were reported in published studies by Lee *et al.* [154] and Kitikoon *et al.* [153]. Representatives of the major IAV-S vaccine manufacturers have expressed willingness to supply interested diagnostic laboratories with monovalent or polyvalent antisera induced by their vaccines. The serological method requires isolation and propagation of virus from the herd, and therefore is not possible if diagnosis is by PCR only, or if the strain in question does not grow well in the laboratory.

### 7.4. Keys to Better-Matched and Innovative IAV-S Vaccines in the Future

Significant improvement in IAV-S vaccines in the longer term will require coordinated efforts by the various parties participating in field surveillance, strain selection, manufacturing, and product licensure. If IAV-S strains in the conventional inactivated vaccines could be updated more rapidly, in response to new trends in the surveillance data, the vaccines would have more consistent efficacy in the field. In Section 7.1 we discussed some practical factors that make it difficult to simply copy the human seasonal influenza vaccine strain selection program that is guided by the World Health Organization. However, veterinary vaccine experts have outlined long-range visions of a more flexible, coordinated, and data-driven program to select IAV-S strains [150,159]. Multi-agency cooperation would need to include manufacturers, NAHLN laboratories, and the relevant agencies within USDA. Cooperation could be focused on several aims:

- Continuous evaluation of circulating strains and their antigenic similarity to current vaccines. The USDA’s program for influenza surveillance in swine should continue to monitor contemporary viruses identified in cases of respiratory disease outbreak, tracking the frequency of subtypes and the emergence of new variants. Representative viruses from prevalent subtypes or genotypes should be evaluated at an antigenic level by serologic cross-reactivity with vaccine anti-sera. Challenge experiments in pigs could be conducted to test for loss of vaccine efficacy when antigenic changes are identified.
- Strain update working group. Manufacturers currently perform independent serology and *in vivo* challenge experiments, at significant cost, to assess if their polyvalent inactivated vaccines provide enough protection against circulating IAV-S strains. If the serology, bioinformatics analysis, and challenge experiments were performed under USDA leadership, it should be a more efficient use of resources.
- Engineering of versatile “donor virus” strain(s) that confer superior manufacturing properties.Some IAV-S strains with desired antigenic properties are limited by poor growth properties, making them difficult to manufacture. As discussed elsewhere in the paper, antibody targets are mainly in HA and NA genes while efficient replication and protein expression in culture tend to be controlled by the internal proteins. Traditional gene reassortment or modern reverse genetics techniques can be used to combine HA and NA genes of a new strain of interest with internal genes of a high-growth strain, making an optimal seed virus for manufacturing. If a donor strain could be qualified in advance, receive regulatory approval, and be made available to all manufacturers, it could speed the process of making strain updates.
- Revise regulatory policies to accommodate new vaccine platforms, such as LAIV and recombinant viral vectored products.

## 8. Autogenous Influenza Vaccines for Swine

Autogenous vaccines are intended for use in situations where commercial vaccines are ineffective or unavailable [160]. Before using an autogenous vaccine, two questions to consider are whether commercial vaccines have been employed and found ineffective, and whether other barriers to vaccine efficacy, such as management factors, exist in the herd and could be contributing to the apparent lack of efficacy [161]. A significant number of U.S. swine farms vaccinate their animals with autogenous IAV-S vaccines. These vaccines were used by 20% of the U.S. farms that vaccinated breeding sows in 2006 [39], and in 2008, they accounted for more than half of all IAV-S vaccine doses released for sale (personal communication in [162]). The primary reason for their use is thought to be the diversity of IAV-S variants circulating among pigs in North America, combined with the limited number of IAV-S strains available in commercial vaccines [39].

### 8.1. Regulatory Requirements for Autogenous Vaccines

In the U.S., autogenous vaccines are regulated by the USDA Center for Veterinary Biologics (CVB). These vaccines must be inactivated, nontoxic, and used only by a veterinarian under a veterinary client/patient relationship [163]. Ordinarily, the vaccine organism(s) must come from the herd in which the vaccine is to be used [163]. Multiple isolates from the herd are commonly used in an autogenous vaccine. In addition to vaccine components from other pathogens, autogenous IAV-S vaccines may contain various combinations of H1 and H3 viruses [162].

An autogenous vaccine is only permitted for use in the herd of origin, unless there is epidemiological evidence that adjacent or nonadjacent herds are at risk [163]. In this case, authorization can be secured from CVB, through a routine process consistent with 9 CFR 113.113.The State Veterinarian, or equivalent state official, must be informed when an authorized vaccine is used in such herds. Autogenous vaccines are tested for safety in laboratory animals (mice or guinea pigs) and for purity [163]. Purity testing includes testing for the absence of viable bacteria and fungi. Autogenous influenza vaccines must also meet the general requirements for the production of viral vaccines [163,164]. These requirements [164] stipulate that tests must be incorporated into the manufacturing process to ensure complete viral inactivation. However, testing is overall less rigorous than required of commercial IAV-S vaccines [162]. In particular, manufacturers of autogenous vaccines do not need to conduct potency and efficacy testing. As a result, some vaccines might contain antigen doses that are suboptimal for that particular viral strain. The expiration date for formulated autogenous vaccines is a maximum of 18 months after harvest [163].

A microorganism can be used for autogenous vaccine manufacturing until 15 months after it was first isolated, or for 12 months after the date of harvest for the first serial [163]. Extensions to 24 months are now permitted without CVB review, provided the firm maintains records documenting, among other things, that the vaccine was beneficial and that the microorganism remains associated with disease in the herd [163,165]. With additional tests and CVB authorization, additional serials may be produced after 24 months has passed since virus isolation [163].

### 8.2. Selection and Use of Autogenous Vaccines

Advantages of autogenous vaccines include the ability to customize the vaccine to the specific virus(es) in the herd, to include multiple agents, and to customize the adjuvant [166]. When selecting a multi-agent autogenous vaccine, it should be noted that there is no requirement to test for potential interference between vaccine antigens or adjuvant components [166]. Given the less rigorous testing requirements, an autogenous vaccine for a new viral strain can be produced and delivered much more rapidly than an updated commercial vaccine. There is nevertheless a significant lag period to isolate and characterize the agent and manufacture the vaccine, compared to ordering a commercial vaccine that is already available. While the absence of extensive testing may also lower the price of autogenous vaccines, this comes at the cost of potential uncertainties in potency and efficacy. In addition, there is an increased risk that adventitious agents might be present in a vaccine prepared from field viruses and not tested extensively [166]. In addition, not all IAV-S isolates adapt well to cell culture, or grow to high titers for vaccine production.

When selecting an autogenous vaccine, consideration should be given to the choice of manufacturer, as the products produced by different companies may vary. Companies that produce USDA-approved commercial biologicals will most likely use similar quality control/quality assurance production standards for their autogenous vaccines [166]. A complete economic evaluation, including performance, morbidity and mortality, can help determine the cost savings or loss from vaccine use; the selection of a vaccine should not be based solely on price [166]. A vaccine trial can help evaluate whether the commercial or autogenous vaccine is more effective in the herd [166]. It is highly recommended that post-vaccination sera be collected to test against the herd IAV-S strains contained in the autogenous vaccine for serologic evidence to support vaccine efficacy.

The efficacy of autogenous vaccines against IAV-S has not been evaluated and reported to a significant extent in the literature. One recent experiment assessed the efficacy of a bivalent autogenous vaccine containing β- and γ-cluster H1 IAV-S, as well as two commercial vaccines, against pandemic H1N1 viruses [162]. All three vaccines provided partial protection against this virus, although all were less effective than an experimental vaccine containing a virus identical to the challenge strain. It is likely that autogenous and commercial vaccine efficacy will differ between herds, depending on the specific viruses that affect those herds.

## 9. Vaccine Associated Enhanced Respiratory Disease (VAERD)

Certain vaccines for other mammalian viruses have been shown to induce immune responses that later exacerbate the severity of infection [167]. With influenza viruses, vaccine failure has usually been equated with little or no protective immunity and lack of prevention of virus transmission in the herd rather than disease exacerbation. However, *in vivo* experiments in recent years have revealed a potentially important phenomenon of vaccine associated enhanced respiratory disease (VAERD) in IAV-S infected pigs. VAERD has been observed in pigs immunized with an adjuvanted, whole inactivated, monovalent IAV-S vaccine and later challenged with an antigenically divergent strain still of the same subtype [38,83,147].

### 9.1. VAERD Pathogenesis

The best studied experimental model of VAERD is in weanling piglets that are vaccinated with δ-1 cluster H1N2 virus and then challenged with 2009 pandemic H1N1 virus [38], or vice-versa [168]. Serum IgG antibodies induced by the vaccine bind to the heterologous challenge virus strain, but provide no significant cross-neutralization. Pigs affected by VAERD sometimes present with more pronounced clinical signs (e.g., fever, dyspnea, coughing, lethargy) compared with non-vaccinated control pigs challenged with the same virus [38,169], but clinical signs are not always significantly exacerbated [83]. Macroscopic pneumonia is more extensive in VAERD pigs’ lungs than in non-vaccinates. Histological examination of VAERD-affected lung reveals more prominent bronchiolitis, peribronchiolar lymphocytic cuffing, and interstitial pneumonia [38,169]. IAV-S may also be more widely distributed in the lungs of pigs with VAERD than in non-vaccinated, challenged animals [83].

VAERD may be the result of multifactorial mechanisms, including dysregulation of proinflammatory cytokines and immune cell types. However, one consistent component seems to be vaccine-induced cross-reactive IgG antibodies in the absence of HI or neutralizing antibodies [38,83,169]. Such cross-reactive IgG antibodies might activate inflammatory immune mechanisms that damage tissues without efficient control of virus replication. *In vitro* assays with serum from VAERD-affected pigs showed evidence that these IgG antibodies bind to the heterologous strain but fail to neutralize it and instead promote virus infectivity and fusion, thus leading to elevated replication [170].

### 9.2. Alternate Immunization Methods and VAERD

Based on experimental data, initial exposure to a live virus does not promote VAERD. In one study, pigs that were inoculated with live, wild-type virus were partially protected from heterologous challenge infection, whereas the group that received a whole inactivated virus vaccine of the same strain went on to develop VAERD after challenge [83]. A different study tested whether a live attenuated influenza virus (LAIV) vaccine presenting the same H1N2 surface antigens as the whole inactivated virus vaccine would also potentiate VAERD after heterologous 2009 pandemic H1N1 challenge [171]. In contrast, the LAIV vaccine conferred significant cross-protection against viral replication and disease. The pronounced difference between inactivated and live vaccines likely results from superior induction of cell-mediated and mucosal immunity with the LAIV vaccine. This further supports the idea that LAIV vaccines could be an effective tool to defend against diverse IAV-S strains.

An additional recent study tested whether VAERD would occur in piglets that received maternal antibodies from whole inactivated virus (WIV)-vaccinated sows, prior to challenge with the heterologous virus [172]. Mismatched antibodies from the mother were sufficient to prime these piglets for VAERD. This result points to IgG antibodies as the immune mediator mainly responsible for triggering VAERD (as opposed to T lymphocytes), and also raises a note of caution about immunizing breeding sows with WIV vaccines if antigenic relevance of vaccine strains is in doubt.

VAERD-like disease has also been reported in pigs immunized with one experimental DNA vaccine and challenged with an H1N1 IAV-S strain. These vaccines encoded M2e (the highly conserved, extracellular domain of the M2 protein) and induced non-neutralizing antibodies against it [32]. The relevance of this experiment to VAERD induced by inactivated virus vaccines is uncertain, although it is possible that the underlying mechanisms causing lung lesions are similar.

### 9.3. Potential for VAERD in Vaccinated Herds

To our knowledge, VAERD has not yet been definitively demonstrated in IAV-S-infected swine herds, although it is inherently difficult to make such a determination under field conditions. Conventionally licensed and autogenous vaccines used in the field contain inactivated viruses with adjuvant, and many circulating IAV-S strains are antigenically distinct from the vaccines, so it is a realistic possibility. On the other hand, vaccines in the VAERD experiments were monovalent, whereas polyvalent commercial vaccines contain antigens from multiple H1 and H3 clusters. Since VAERD pathogenesis requires a major antigenic difference between vaccine and challenge virus (*i.e.*, two viruses of different clusters), pigs receiving a polyvalent commercial vaccine may not be as vulnerable to VAERD. Data from two studies where pigs were vaccinated with multivalent commercial vaccines before challenge with 2009 pandemic H1N1 virus, with no report of VAERD, support this idea [162,173].

Natural infection also differs from the experimental setting in the route of exposure: droplet transmission from animal to animal, *versus* intratracheal or intranasal inoculation. The impact this might have on VAERD is not known. Thus the VAERD studies, like most animal experiments, simplify some of the complex conditions found in the field to make the outcomes more reproducible. It is nevertheless prudent to take vaccine-enhanced disease into account as a possible hazard to herd health, and consider ways to lower that risk.

### 9.4. Concepts to Limit the Potential for VAERD

With the imperfect choices of commercial and autogenous IAV-S vaccines available today, what strategies could reduce the risk of VAERD in vaccinated swine herds? The preference for a vaccine with the best-possible antigenic match, even if it is not a full match, holds true with respect to avoiding VAERD. Ideas mentioned above for optimal protection included comparing gene sequence of field isolates and vaccine seed viruses, or developing antisera to commercial vaccines that would enable serological tests against field strains (Section 7.4). Both of these would be useful ways to identify the most relevant vaccine option and reduce the risk of VAERD. Also, a polyvalent vaccine that contains representatives of multiple H1 and H3 clusters would be less likely to potentiate VAERD than a bivalent vaccine with only one older, antigenically distant strain of each subtype.

Autogenous vaccines provide assurance that the currently circulating strain will be well matched to the vaccine and unlikely to cause VAERD. The drawback is that a new strain belonging to an antigenically divergent cluster could emerge or co-circulate, in which case antibodies already induced by the autogenous vaccine might predispose pigs to VAERD. Perhaps the most stringent way to supply protection and prevent VAERD is to immunize with an autogenous vaccine to control the locally predominant virus plus multivalent commercial vaccine to control other strains that may enter the herd. Finally, experimental data indicate that LAIV vaccines could one day be an excellent tool to provide cross-protection, and a minimal risk of VAERD, during outbreaks with diverse IAV-S strains.

## 10. New Vaccine Technologies for IAV-S

### 10.1. Replicon Particle Vectored HA

Alphavirus-like replicon particles (RP) are the first viral vector vaccine technology approved by USDA as a vaccine for IAV-S (“Swine Influenza Vaccine, RNA”; Harrisvaccines, Ames, IA, USA) [174]. The vector is a modified form of an attenuated Venezuelan equine encephalitis virus, from which structural genes have been deleted, thus preventing multi-cycle replication of the virus (reviewed by Vander Veen *et al.* [175]). In place of deleted genes, it is possible to package RNA that encodes a gene of interest, such as the HA gene of an IAV-S strain. The licensed RP IAV-S vaccine includes a sequence encoding the HA of a cluster IV H3N2 virus. Formation of the propagation-defective particles requires separate strands of RNA (helper RNAs) that are engineered to express the structural genes for only that initial cycle. A recombinant alphavirus RP then has the capacity to bind host cells, deliver genetic cargo to the cytoplasm, and drive protein expression of the inserted gene(s), without producing any progeny virus [175]. Alphavirus RPs also have inherent adjuvant properties [176], which likely contributes to effectiveness as a vaccine platform.

*In vivo* testing of RP-HA vaccines in pigs, using H1 or H3 genes of multiple IAV-S strains, demonstrated their ability to induce robust titers of HA inhibiting (HI) antibodies [177,178,179]. Pigs vaccinated with two doses of RP-HA vaccine (H3 subtype) were protected against homologous H3N2 challenge infection, in terms of nasal shedding of virus, gross lung lesions, and histological lung scores [177,180]. Anti-vector immunity against alphavirus RPs is considered to be limited, due to the deleted structural genes, which suggests booster doses can be administered without interference by circulating antibodies [181]. One might also predict that RP-HA vaccines can resist interference from prior IAV-S immunity, including maternal antibodies, but a study in maternal antibody-positive piglets showed clear interference with immune responses to RP-HA vaccination [177]. Since HA is the only influenza gene encoded by this vaccine, there is no antibody response directed against other influenza proteins, such as NA, NP, or M1, which may diminish the partial immunity afforded by whole virus vaccines in the absence of a well-matched neutralizing response to the HA. However, the vaccine platform is well-suited to diagnostic strategies of differentiating infected from vaccinated animals (DIVA). Another advantage of the RP-HA vaccine system is its adaptability for making rapid IAV-S strain updates. By routine modern methods, the HA gene from any emerging or newly dominant strain can be cloned (or synthesized from scratch, based on published sequence) and inserted into the RP vector [179]. This enables rapid establishment of new replicons for vaccine manufacturing.

The replicon particle vaccine platform that was the basis for licensure of “Swine Influenza Vaccine RNA” vaccine, identified as SirraVax™ technology (Harrisvaccines, Inc., Ames, IA, USA), is more frequently being used by Harrisvaccines to generate custom IAV-S vaccines. Each custom RP HA IAV-S vaccine is designed based on HA gene sequence(s) submitted in an electronic format by a veterinarian. A nucleic acid molecule is synthesized to encode the same HA protein sequence. Vero cell cultures are transfected with the recombinant replicon RNA and helper RNA, using electroporation, and incubated for a designated period of time. Harvested RP-HA is then purified by affinity chromatography and formulated, without an adjuvant. Pork producers that have identified multiple distinct strains may submit several HA sequences, enabling the production of polyvalent custom RP-HA vaccines. Combination products blending the RP-HA with custom RP-vectored antigens of other establishment-specific viruses, such as rotavirus variants, are also possible. The custom manufacturing process typically takes 4–6 weeks. These custom vaccines function essentially in the same way as autogenous vaccines. To date, a USDA autogenous vaccine license has not been issued for SirraVax™-based custom RP-HA influenza vaccines. Harrisvaccines produces and sells these products without a USDA license, citing the veterinary practitioner exemption (9 CFR, Part 107.1).

### 10.2. Adenovirus Vectored IAV-S Genes

Replication-defective human adenovirus serotype 5 (Ad5) is a viral vector strategy with a significant experimental track record in swine. Deletion of a portion of this virus’s genome created a replication defective phenotype, as well as space in the genome to insert foreign genes. Replication defective Ad5 can only be grown in specialized cell lines with genes that compensate for the deleted gene region of the virus.

The HA and NP genes of an H3N2 IAV-S strain were both cloned into Ad5 to generate separate recombinant viruses, which were then tested, together or individually for immunogenicity and vaccine efficacy in pigs [182]. Single-dose intramuscular delivery of Ad5-HA, with or without the Ad5-NP construct, elicited HI antibodies. Pigs given Ad5-HA alone were protected moderately well against heterologous H3N2 virus challenge, while the combination of Ad5-HA and Ad5-NP supplied more complete protection, in terms of viral replication and lung lesion severity. One of the subsequent studies with this vaccine model showed that the Ad5-HA plus Ad5-NP antigens could be delivered to muscle tissue via a needle-free system, with similar results to intramuscular injection [183].

It was also shown that the Ad5-vectored IAV-S vaccine can be used effectively in piglets with influenza-specific maternal antibodies. The combined Ad5-HA and Ad5-NP antigens, administered intramuscularly in the face of H3N2-specific maternal antibodies, triggered an active HI antibody response [184]. A boosting dose with commercial inactivated IAV-S vaccine, given 4 weeks later, produced a clear anamnestic antibody response. This group of pigs then showed superior protection against H3N2 challenge infection compared to pigs that received only the inactivated IAV-S vaccine.

Most recently, a recombinant Ad5 encoding HA of the 2009 pandemic H1N1 virus was used to immunize pigs by a single intranasal dose [168]. This immunization induced IAV-S-specific T cells and nasal IgA antibodies, while providing solid protection against homologous challenge. Immune mediators elicited by the vaccine were partially cross-reactive with the heterologous H1N2 challenge virus (δ-1 cluster), supplying a modest level of cross-protection.

Collectively, these data show that the adenovirus vector is a potentially useful system for immunizing pigs against IAV-S. The ability to generate IAV-S immunity in piglets with IAV-S specific maternal antibodies is important, but perhaps partly offset by difficulty boosting animals with Ad5-HA after they have already been primed with the same vaccine [185]. Other desirable factors with Ad5 are the safety of a non-replicating vector and the versatility to respond rapidly to a new epidemic or pandemic by inserting a new, matching HA gene [186]. Commercial development of this strategy is not underway at this time, to our knowledge, but the significant body of data published in the past suggests it could be a valuable vaccine system, perhaps for use in combination with conventional IAV-S antigens.

### 10.3. Live-Attenuated (Modified-Live) Virus

Over the past 10 years, experimental studies of live-attenuated influenza virus (LAIV) vaccines for swine have shown great promise. (LAIV is equivalent to modified-live virus (MLV).) The rationale for LAIV vaccines is that since they are administered by the intranasal route, the attenuated viruses replicate moderately in the upper respiratory tract and induce a balanced mucosal and systemic immune response. Mucosal antibodies in the respiratory tract lining are very desirable in the context of controlling airborne influenza virus infection. The potential for replicating LAIV to induce a robust cell-mediated immune response is also an attractive mechanism for defense against IAV-S because most T cells recognize epitopes in the conserved internal proteins, making them broadly reactive against many strains.

Three different research teams developed LAIV vaccines for IAV-S, each using distinct strategies of viral gene manipulation to cause an attenuated phenotype [36,187,188]. All three designs were made possible with reverse genetics technology, which starts by inserting DNA copies of the eight viral RNA gene segments into separate plasmids that are manipulated using standard molecular biology techniques [189]. A specific reassortant virus can be generated by combining gene segments from two or more viral strains, such as substituting the HA and NA from a contemporary circulating strain onto a lab adapted vaccine backbone strain. Attenuating mutations can be made in one or more gene segments, which then can be combined with HA and NA genes from a vaccine strain, thus yielding a virus with the desired surface antigens plus attenuating properties encoded elsewhere. One of the attenuation approaches was to handicap the virus’s ability to resist host cell type I interferon (IFN), by reducing the length of itsNS1 protein [35,187]. A second approach was to make the virus cold-adapted and temperature sensitive by changing amino acid residues in two of the viral polymerase genes, very similarly to the licensed human LAIV vaccine (FluMist) [36]. A third approach modified the cleavage site in HA to require elastase enzyme; elastase can be added as a supplement during virus cultivation, but it is too scarce in the host animal to support significant replication of the virus [188].

Independent studies tested all of these vaccine platforms in pigs, using two intranasal doses, for immunogenicity and efficacy against infection. Summarizing briefly, the LAIV vaccines proved immunogenic in terms of eliciting respiratory tract mucosal antibodies, systemic cell-mediated immune responses, and modest levels of systemic neutralizing antibodies [34,35,36,190]. In terms of efficacy, all of the experimental LAIV vaccines have shown robust protection against challenge with homologous virus, plus measurable and sometimes robust cross-protection against antigenic variant virus. An LAIV vaccine proved efficacious against infection with an antigenically distinct challenge strain, even if the vaccine was administered to piglets with high levels of maternal antibodies [191].

A key factor in terms of the cost and convenience of vaccine application in the field is whether two doses are required (two doses are required with conventional inactivated vaccines). In one study, piglets receiving a single dose of NS1 mutant LAIV developed cross-protective immunity comparable to piglets that received two doses [191]. Because sow vaccination represents a large part of the IAV-S vaccine market, another study explored the use of temperature sensitive H1N1pdm09 LAIV in sows [172]. Piglets from litters that suckled colostrum from LAIV-vaccinated mothers received partial protection against homologous challenge, but not against heterologous δ1-H1N2 challenge.

These studies point to LAIV vaccines as a platform with potential to address two of the critical problems in IAV-S immunization: antigenic variation and interference from circulating maternal antibodies. Another problem that has been linked to whole inactivated virus vaccines, at least in experimental challenge studies, is VAERD. When LAIV and inactivated vaccines of identical strains were administered to piglets, followed by heterologous challenge, LAIV vaccinees showed none of the VAERD phenomenon present in WIV vaccinees, but were in fact partially cross-protected [171]. Therefore, it can be envisioned that an LAIV vaccine may be successfully developed and licensed, as an equivalent multivalent product has been licensed for humans since 2003 [192]. Regulatory concerns (e.g., safety and environmental impact) and designing a practical mechanism for intranasal mass-vaccination are among the hurdles that would have to be overcome.

### 10.4. DNA Vaccines

DNA plasmid vaccines that encode protective antigens are an attractive concept for veterinary medicine, including IAV-S immunization. A DNA vaccine offers a way to induce expression of proteins of interest in the host animal’s cells, which is a key for inducing robust cell-mediated immunity, yet without exposing the host to any pathogens. The platform enables versatile combination of antigens from multiple strains and simple substitution of one strain for another. Maternal antibodies are considered less likely to impede DNA vaccination, compared with injections of protein antigens or attenuated virus.

DNA vaccines against several swine viral pathogens have been tested in experimental settings. A number of these studies have demonstrated immune responses and protection against challenge infection, including IAV-S [193,194,195]. HA-encoding DNA vaccines for IAV-S were administered successfully by intramuscular needle injection as well as a needle-free subcutaneous/intramuscular injection method [196]. The major drawback with these proof-of-concept studies is that large doses of DNA were required, often in a series of three or more doses. Because materials and labor costs to administer these doses would be high, DNA vaccines for IAV-S are probably not commercially viable, with current methods.

### 10.5. Subunit Vaccines

Viral subunit vaccines contain protein antigens that are either fractionated from whole virus or expressed individually in a recombinant system. A subunit vaccine consists of a simpler protein mixture than whole inactivated virus, which allows the target proteins (such as HA) to be formulated at a greater concentration and purity. Most human seasonal influenza vaccines in North America and Europe are subunit vaccines derived by treating virus with detergent and isolating the membrane proteins (mainly HA and NA) [149].

One recently licensed human seasonal influenza vaccine is a subunit product called FluBlok, which is comprised of recombinant HA (rHA) proteins expressed from a recombinant baculovirus vector [197]. The rHA proteins of different strains are produced in insect cell culture, purified, and blended to form a trivalent final product. The baculovirus-vectored expression system should be equally capable of generating IAV-S vaccine antigens. In addition, the alphavirus RP vector system can be utilized to express high levels of rHA protein in mammalian cell culture [179,198]. (This differs from the alphavirus RP vaccine approach discussed in Section 10.1, which delivers recombinant RP-HA into the animal, leading to *in vivo* expression of the rHA protein.) Other culture systems that have been engineered to express immunogenic rHA include *E. coli*, yeast, and plants. Subunit vaccines for IAV-S might offer advantages in terms of production efficiency and the flexibility to substitute strains. It is unlikely that subunit IAV-S vaccines would differ very much from conventional inactivated virus vaccines in terms of efficacy, but they would be suitable for developing DIVA diagnostics.

## 11. Conclusions

IAV-S became a much more costly and complicated problem for the US pork industry after the triple reassortant virus lineage emerged in the late 1990s and began undergoing gene reassortment with various other swine and human influenza viruses. Since that time the antigenic diversity of circulating strains has increased and the field efficacy of conventional IAV-S vaccine formulations has been increasingly questioned. The conventional IAV-S vaccines, which contain inactivated viruses representing up to 5 strains, are designed to induce robust titers of serum neutralizing antibodies. However, the degree of cross-neutralization against antigenically drifted strains is often very limited. Some experimental results suggest that vaccine-induced antibodies can even enhance the severity of subsequent infection with a poorly matched strain.

The efficacy of conventional vaccines would be greater if IAV-S strains were updated more frequently to improve the likelihood of antibodies matching the prevalent circulating strains. The USDA IAV-S surveillance program that was launched in 2010 generates data that could support a coordinated system of vaccine strain selection. Practitioners seeking to identify the best-matched vaccine for an IAV-S strain in a client’s herd can utilize gene sequencing and/or serology to analyze the similarity between that field virus and the strains used in each of the commercial vaccines. Resulting data may indicate there are no closely matching commercial vaccines, and that an autogenous or custom vaccine is a better strategy. Experimental research shows promising results for new forms of IAV-S vaccines, such as live-attenuated or recombinant viral vectors, which may at some point offer a more broadly-protective method of immunizing pigs. Vaccines that reliably prevent IAV-S outbreaks in swine would be expected to improve the efficiency of pork production while reducing the danger of virus transmission to humans.
